# Cardiovascular Magnetic Resonance-Based Three-Dimensional Structural Modeling and Heterogeneous Tissue Channel Detection in Ventricular Arrhythmia

**DOI:** 10.1038/s41598-019-45586-1

**Published:** 2019-06-27

**Authors:** Jihye Jang, Hye-Jin Hwang, Cory M. Tschabrunn, John Whitaker, Bjoern Menze, Elad Anter, Reza Nezafat

**Affiliations:** 1 Department of Medicine, Beth Israel Deaconess Medical Center and Harvard Medical School, Boston, MA USA; 2 Department of Computer Science, Technical University of Munich, Munich, Germany; 3 Division of Cardiovascular Medicine, University of Pennsylvania, Philadelphia, PA USA; 4 Division of Imaging Sciences and Biomedical Engineering, King’s College London, London, United Kingdom

**Keywords:** Translational research, Risk factors, Predictive markers, Cardiology, Ventricular tachycardia

## Abstract

Geometrical structure of the myocardium plays an important role in understanding the generation of arrhythmias. In particular, a heterogeneous tissue (HT) channel defined in cardiovascular magnetic resonance (CMR) has been suggested to correlate with conduction channels defined in electroanatomic mapping in ventricular tachycardia (VT). Despite the potential of CMR for characterization of the arrhythmogenic substrate, there is currently no standard approach to identify potential conduction channels. Therefore, we sought to develop a workflow to identify HT channel based on the structural 3D modeling of the viable myocardium within areas of dense scar. We focus on macro-level HT channel detection in this work. The proposed technique was tested in high-resolution *ex-vivo* CMR images in 20 post-infarct swine models who underwent an electrophysiology study for VT inducibility. HT channel was detected in 15 animals with inducible VT, whereas it was only detected in 1 out of 5 animal with non-inducible VT (P < 0.01, Fisher’s exact test). The HT channel detected in the non-inducible animal was shorter than those detected in animals with inducible VTs (inducible-VT animals: 35 ± 14 mm vs. non-inducible VT animal: 9.94 mm). Electrophysiology study and histopathological analyses validated the detected HT channels. The proposed technique may provide new insights for understanding the macro-level VT mechanism.

## Introduction

The geometric structure of the myocardium plays an important role in the generation of cardiac arrhythmias. Narrow anatomical structures cause slow conduction and generate arrhythmias in the cavotricuspid^1^ or mitral isthmuses^2^. In infarcted hearts, surviving tissues within the scar create heterogeneous tissue structures that cause slow conduction^3^ and contribute to arrhythmogenesis.

The importance of myocardial structure in arrhythmias has led cardiovascular magnetic resonance (CMR) to a promising technique for identifying the arrhythmogenic substrate. Late gadolinium enhancement (LGE) CMR allows accurate imaging of the extent and location of the myocardial infarction^4,5^ and can identify the substrate in scar-related ventricular tachycardia (VT). Studies have shown that the scar border/heterogeneous zone defined by LGE are associated with VT inducibility^6^ and spontaneous ventricular arrhythmias^7–9^.

Studies have identified conduction channels by CMR and correlated them with those defined by electroanatomic mapping^10–16^. In the *in-vivo* setting, multiple 2D slices of LGE images or endo-to-epi myocardial shells of LGE have been reviewed to identify conduction channels^13,15,16^. In the *ex-vivo* setting, where higher-resolution imaging is possible, 3D reconstruction of the myocardial scar has been performed to identify conduction channels^10,11^. However, these data are mainly based on qualitative identification of the channels without a streamlined workflow. That is, readers may identify conduction channels differently given identical LGE scar/myocardial images. Furthermore, histological validation in these data is limited.

Despite the potential of CMR to characterize the arrhythmogenic substrate, there is currently no standard approach to identify potential conduction channels. Therefore, this study seeks to develop a workflow to identify heterogeneous tissue (HT) channels, defined as narrow pathways consisting of healthy tissue, surrounded by scar or an electrically non-excitable medium (e.g. fat, blood) and connected to healthy myocardium located within areas of dense scarring. The proposed technique was tested in high-resolution *ex-vivo* CMR images in 20 swine models with VT following myocardial infarction, and validated by electrophysiology and histopathological analyses.

## Methods

**Figure 1:**
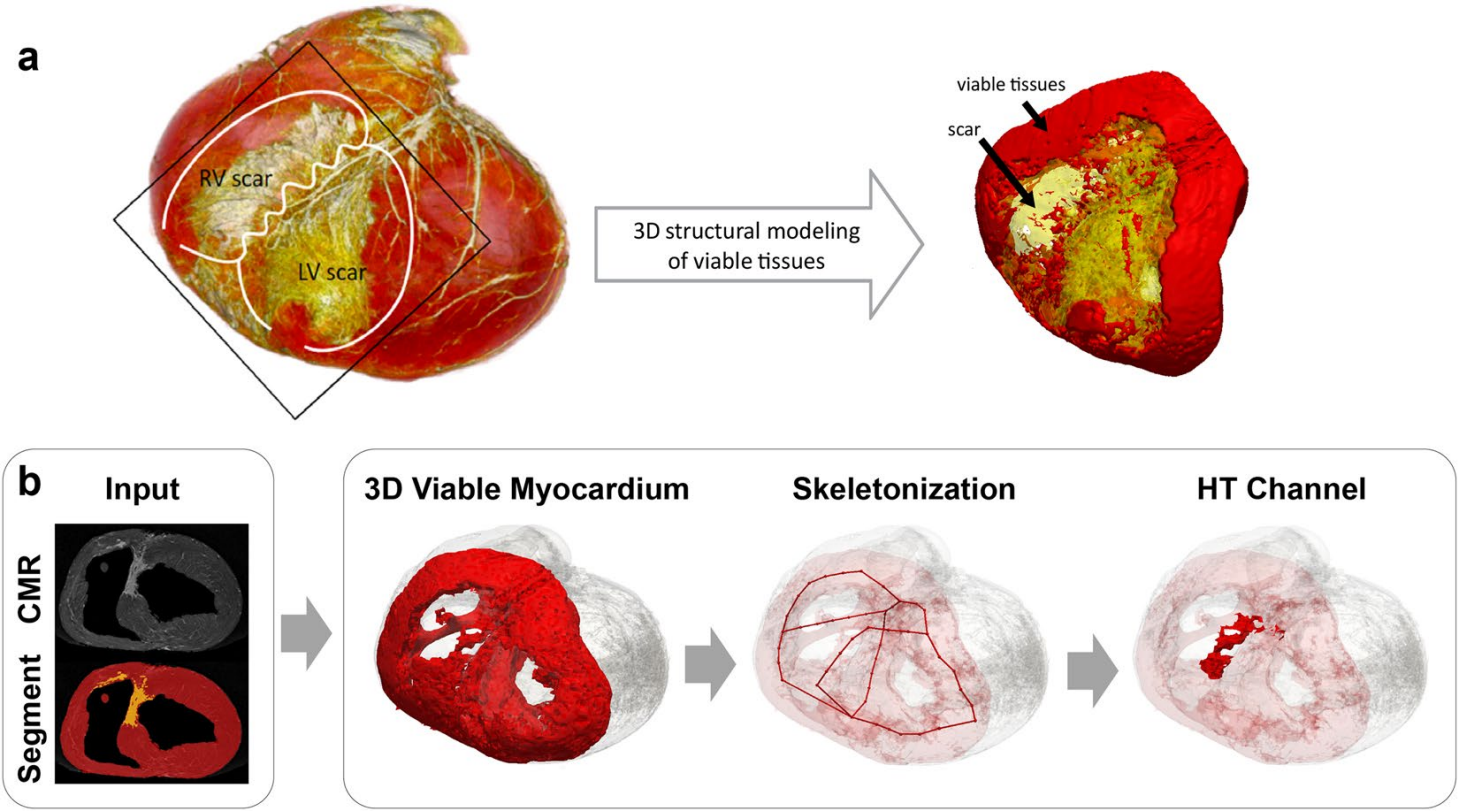
(**a**) A 3D volume rendered view of the 3D *ex-vivo* cardiovascular magnetic resonance (CMR) images (yellow, scar; red, viable myocardium) and 3D structural model of viable myocardium in and around the scar generated with triangular surface meshes (yellow, scar; red, viable myocardium). (**b**) Proposed workflow for detection of a macro-level heterogeneous tissue (HT) channel uses scar and myocardium segmentation from CMR images as input and performs the following steps. Step 1: A 3D structural model of viable myocardium is generated from 3D CMR images to visualize the propagation pathway of a cardiac electrical signal. Step 2: 3D skeletonization of viable myocardium is performed to extract the simplified geometry of the viable tissue and enable automated structural model characterization. Step 3: HT channels are detected based on the 3D skeleton segmental analysis that identify HT channels with a high 3D structural complexity. RV indicates right ventricle; and LV, left ventricle.

We propose a technique for 3D HT channel detection based on 3D structural modeling of the viable myocardium using high-resolution *ex-vivo* CMR (Fig. 1a). We propose a workflow which uses segmentation of the scar and myocardium from CMR images as input and performs automated detection of HT channels (Fig. 1b). In particular, we focus on detecting macro-level HT channels. The overall processing pipeline consists of 3 steps: (1) 3D structural modeling of viable myocardium, (2) 3D skeletonization of viable myocardium, and (3) automated HT channel detection. The first step is to extract viable myocardium within areas of dense scar where a cardiac electrical signal propagates. The second step is to simplify the geometry of the viable myocardium and enable automated characterization of the structures of the viable myocardium model. The last step is to identify HT channels based on the 3D structural properties of the viable myocardium. Each step is detailed in the following sections.

### 3D structural modeling of the viable myocardium

**Figure 2:**
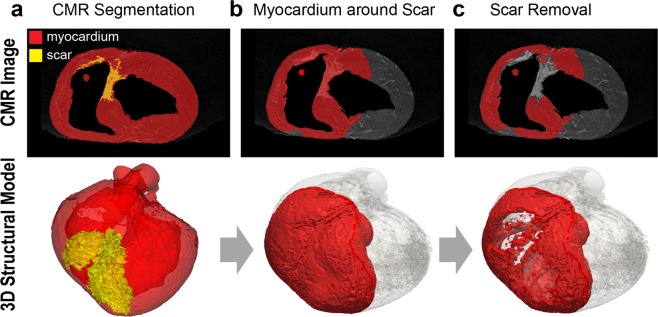
The proposed pipeline for building the 3D structural viable myocardium model. Upper panels show *ex-vivo* cardiovascular magnetic resonance images with overlaid myocardial segmentation (red), and the 3D structural model view of the corresponding segmentation (red) is represented in the lower panels. The whole ventricular 3D volume is shown in transparent gray. (**a**) Scar and myocardium segmentation are used as an input to the system. (**b**) Myocardium in and around the scar was extracted by expanding the 3D volume of the segmented scar to include neighboring myocardium. (**c**) The scar was removed from the myocardium to exclude scarred tissues and to focus only on the viable myocardium where electrical signals propagate. Scar volume was expanded 1 mm before subtracting to remove partial volume errors.

Viable myocardium in and around the scar border plays an important role in VT substrate detection. To explore structural characteristics of viable myocardium within dense scar regions, we performed 3D structural modeling using high-resolution *ex-vivo* CMR images. An experienced reader performed scar and myocardial segmentation from CMR images (Fig. 2a). A second reader independently performed scar/myocardial segmentation in a subset of 6 animals to assess inter-observer variability. Given our interest to extract surviving myocardium near the scar, the first step was to perform a spatial growth operation to localize the myocardial volume neighboring the scar (Fig. 2b). We extracted any myocardium 30 mm isotropically within the scar region as a good trade-off between skeletonization and discrimination of normal myocardium (Supplementary Fig. 1). The segmented scar was then removed from the myocardial volume to extract only viable myocardium around the scar (Fig. 2c). Before subtraction, the scar volume was expanded 1 mm isotropically to remove partial volume errors. The aforementioned processing was performed using the 3D Slicer^17^.

### 3D skeletonization of the viable myocardium

**Figure 3:**
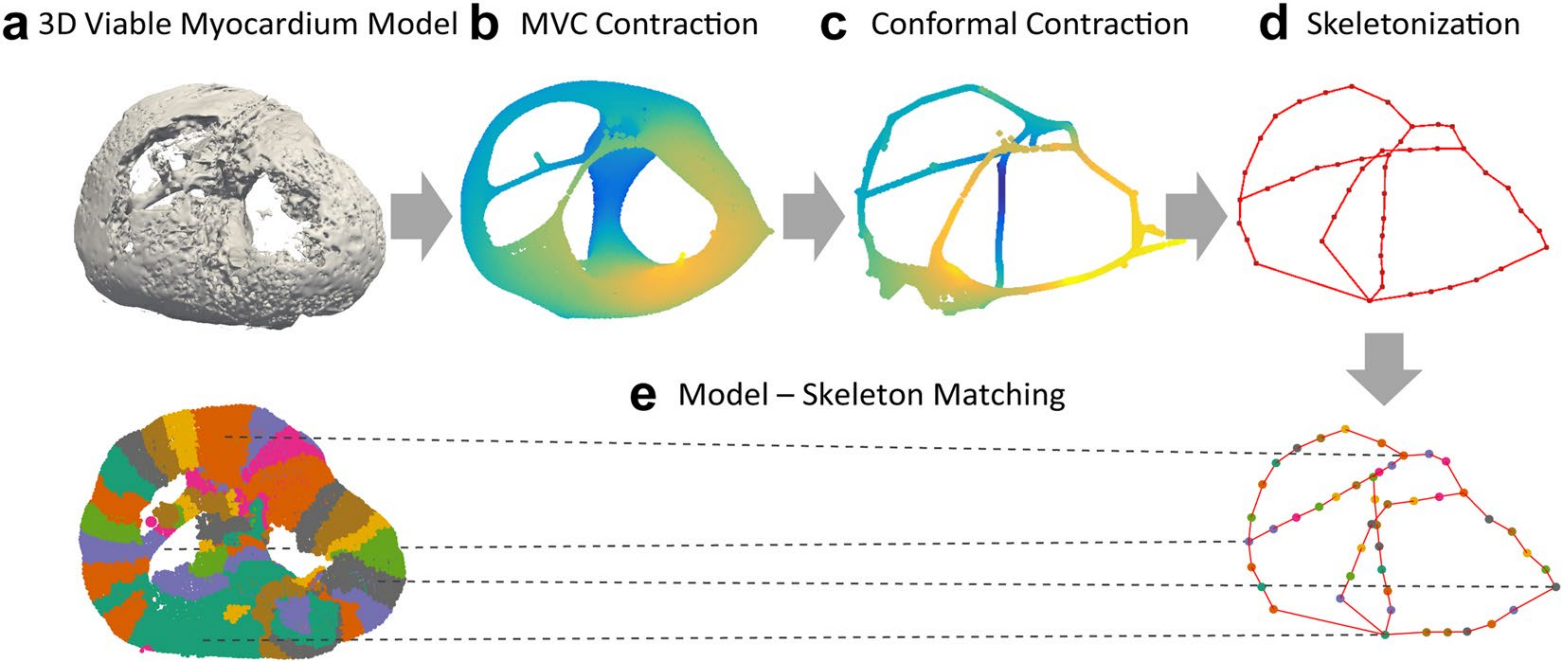
The proposed pipeline for 3D skeletonization to simplify viable myocardial geometry and enable automated quantification of the 3D viable myocardium model. (**a**) Laplacian-based maximal voluntary contraction (MVC) (**b**) and conformal contraction (**c**) were performed to abstract and smooth the shape of the complex structures of the 3D model. 3D skeletonization was performed by topological thinning, resulting in a 3D skeleton with its simplified geometry represented by lines. (**d**) During the skeletonization process, each segment of the structural model is contracted as a point on the 3D skeletal graph, so that the structural properties of the model can be automatically characterized using the skeletal graph (**e**).

3D skeletonization was performed to simplify the geometry of the complex viable myocardial structures and enable automated characterization of the 3D structural model. Before performing 3D skeletonization, the 3D structural model was first reduced to a smaller number of faces and vertices to reduce processing time and memory allocations (Fig. 3a), followed by removal of duplicate faces. As we are interested in the connected pathway of cardiac conduction through viable myocardium, any non-connected isolated myocardium less than 100 triangles, typically considered noise, was removed (Supplementary Fig. 2). A curved 3D skeleton was then extracted from the 3D viable myocardium model using Point Cloud Skeletons via Laplacian-Based Contraction^18,19^. 3D models were first normalized such that all models were located at the center of unit-sized cubes. Point cloud contraction was first performed to smooth and contract the 3D model into an approximate zero-volume mesh that abstracts the given shape and topology. The Laplacian-based maximal voluntary contraction (MVC) was first performed via local Delaunay triangulation (Fig. 3b), which was further contracted using the Conformal Laplacian operation (Fig. 3c). A final 3D skeletal graph consisting of points (nodes) and connecting lines (edges) was created by topological thinning (Fig. 3d). During the skeletonization process, each segment of the structural model was contracted into nodes of the 3D skeletal graph, and the connection of each segment was represented as connecting edges of the 3D skeletal graph (Fig. 3e).

### Heterogeneous myocardial channel detection

**Figure 4:**
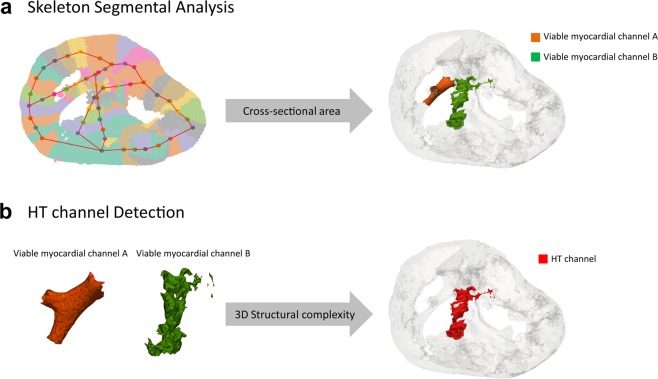
The proposed algorithm for automated heterogeneous tissue (HT) channel detection. The algorithm consists of two steps: (1) skeleton segmental analysis of the cross-sectional area to detect narrow and connected segments to identify viable myocardial channels, and (2) HT channel detection based on the 3D structural complexity to detect HT channels which are known to cause slow conduction. (**a**) For each skeletal point, its orthogonal axis is computed based on its two adjacent nodes, and the cross-sectional plane is calculated. Any connected segments with a cross-sectional area less than 90 mm^2^ are automatically detected to identify narrow strips of tissue. (**b**) Detected myocardial channels are further evaluated for tissue heterogeneity based on the 3D structural complexity defined as the unit number of triangular faces. Any myocardial channel with a heterogeneity index (unit number of triangular faces) greater than 26 was identified as an HT channel.

A skeletal graph enables automated characterization of structural properties of each segment and their connectivity. An anatomic or structural HT channel was defined as a narrow pathway consisting of healthy tissue surrounded by scar or an electrically non-excitable medium and connected to healthy myocardium. HT channel detection consisted of two key steps. First, any narrow tissue channel strip, defined as connected thin tissue segments, were detected. For each node of the skeletal graph, its orthogonal axis was computed based on the direction of its two adjacent nodes, and its cross-sectional plane was calculated. Skeletal node segments with a cross-sectional area less than 90 mm^2^ were automatically detected to discriminate narrow channels from normal myocardium, as determined by the typical thin right ventricle (RV) wall thickness ([90 mm^2^] ≈ [3 mm minimal RV wall thickness in normal^20,21^] × [30 mm neighboring volume expansion (Supplementary Fig. 1)]) (Fig. 4a). The minimal number of connected segments was set to 3 to minimize detection of normal myocardium segments (left ventricle (LV), RV, and septum) (Supplementary Fig. 3a). Second, viable myocardial channels with high 3D structural complexity were detected. As heterogeneous myocardium is often a mixture of preserved and scarred tissue, it forms a complex 3D structure compared to smooth normal tissue. Structurally complex objects require a higher number of faces to model the finer details. Therefore, we defined the HT index as the number of triangular faces per unit length (mm) (Fig. 4b). The HT index was set to 26 to discriminate smooth vs. heterogeneous myocardial tissue channels (Supplementary Fig. 3b). The proposed algorithm was implemented in MATLAB (MathWorks, Natick, MA).

### Animal study

An animal study was performed to test the proposed techniques in swine with scar-related VTs using high-resolution *ex-vivo* CMR images in 20 animals. The study was carried out in strict accordance with the recommendations in the Guide for the Care and Use of Laboratory Animals of the National Institutes of Health. The protocol was approved by the Institutional Animal Care and Use Committee (Protocol Number: 100-2014). All experiments were performed under general anesthesia with isoflurane inhalation (1.5–2.5%) and mechanical ventilation (12–16 breaths/min with tidal volumes between 300–400 ml), and animals were euthanized with pentobarbital sodium.

#### Animal model

Twenty pigs (30–35 kg) underwent 180-minute balloon occlusions of the mid-left anterior descending (LAD) artery as previously described^22,23^. An angioplasty balloon was inflated in the mid-LAD under fluoroscopic guidance. After 180 minutes, the balloon was deflated and withdrawn, in order to create an ischemia-reperfusion mediated myocardial infarction.

#### CMR study

CMR imaging was performed using a 1.5 T scanner (Philips Achieva, Best, The Netherlands) with a 32-element cardiac phased-array receiver coil. Upon completion of the electrophysiology study, the animal was euthanized and the heart was explanted. An intravenous injection of 0.15–0.2 mmol/L gadobenate dimeglumine was performed 15 minutes before euthanasia. Atria was excised and ventricles were filled with kinetic sand to maintain basic geometry. Scar imaging was performed using a high-resolution 3D gradient echo sequence. Typical imaging parameters were as follows: spatial resolution = 0.4* × *0.4* × *0.5 mm^3^; FOV = 130* × *130* × *100 mm^3^; TR/TE = 16/7.4 ms; flip angle = 25°; low-high phase-encoding order; signal averaging = 4.

#### Electrophysiology study

All animals underwent electrophysiology study after an 8-week survival period. Percutaneous femoral arterial and venous access were obtained. Under fluoroscopic guidance, a 6Fr pentapolar diagnostic catheter (Bard EP, Lowell, MA) was placed in the right ventricular (RV) apex to allow pacing. The proximal electrode was positioned in the inferior vena cava and served as an indifferent unipolar electrode. Electrical stimulation was performed from the RV apex using a current strength twice the capture threshold and pulse width of 2 ms. Programmed ventricular stimulation at paced cycle lengths of 600 and 400 ms with 1–4 extra-stimuli down to ventricular effective refractory period were performed to induce VT. If electrical stimulation from the RV apex failed to induce VT, stimulation was repeated from the LV endocardium and epicardium near the infarct region until the animal was inducible. Animals were only considered non-inducible if a VT could not be induced despite stimulation from all sites. Sustained monomorphic VT was defined on 12-lead electrocardiogram (ECG) as a tachycardia lasting >30 seconds with a consistent morphology. A macroscopic site of VT origin was identified using a surface 12-lead ECG^24,25^ to compare with the region of detected HT channels. Furthermore, activation mapping during VT was attempted to identify the detailed site of VT origin.

#### Histopathological analysis

After the *ex-vivo* CMR study, the hearts were placed in a 10% buffered formalin solution for >1-week for tissue fixation, then serially sectioned parallel to the atrioventricular groove into 5-mm thick slices starting from the apex. Tissue samples were paraffin-embedded using large tissue histology cassettes. The tissue was sectioned with a 5-*µ*m thickness, and slides were stained with Masson’s trichrome for collagen detection and digitized. The corresponding CMR slides were identified and compared to histological regions of infarction for characterization of myocardial fibrosis and surviving tissue in the region of detected HT channels.

### Statistical analysis

To test the null hypothesis that there are no non-random associations between VT inducibility and the existence of the detected HT channel, Fisher’s exact test was performed. A result was considered statistically significant at P* < *0.05.

## Results

### Animal model

All animals had extensive anterior-septal LGE at 8 weeks post myocardial infarction. VT was inducible in 15 animals. The remaining 5 post-infarct animals were non-inducible.

### 3D structural modeling and HT channel detection

**Figure 5:**
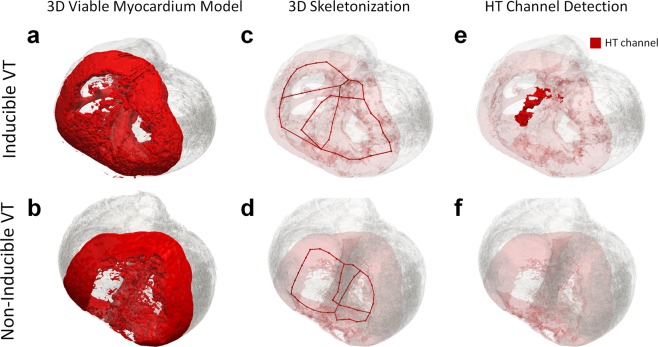
Example of 3D structural viable myocardium models, 3D skeletons, and detected heterogeneous tissue (HT) channel in an animal with an inducible ventricular tachycardia (VT) and an animal with a non-inducible VT. The 3D viable myocardium model was able to represent surviving and viable myocardium in and around the scar in a true 3D manner for each case. (**a**,**b**) 3D skeletonization enabled simplification of the geometry of the viable myocardium. (**c**,**d**) An HT channel was detected in the animal with the inducible VT (**e**), whereas it was not detected in the animal with the non-inducible VT (**f**).

Three dimensional structural modeling enabled visualization of surviving myocardium in and around the scar region in a true 3D manner. Examples of 3D structural models are shown in animals with inducible VTs (Fig. 5a) and non-inducible VTs (Fig. 5b). Three dimensional structures for all animal models are shown in Supplementary Figs 4–7a.

The skeletal graph simplified the complex 3D structure and enabled quantification of structural properties of the 3D model, thereby facilitating automated identification of the HT channels. Figure 5c,d and Supplementary Figures 4–7b show 3D skeletonization results represented by red lines in all animals.

HT channels were detected in all inducible VT animals and highlighted in red (Fig. 5e and Supplementary Figs 4–6c). HT channels were mostly undetected in the animal with the non-inducible VT (Fig. 5f and Supplementary Figures 7c). The examples of the 3D rendered view of the results are shown in Supplementary Videos 1 and 2.

Inter-observer agreement between two independent readers was strong with a Dice index for scar segmentation of 0.79 ± 0.07. HT channel detection results from both readers are shown in Supplementary Fig. 8.

### HT channel and VT inducibility

In 15 animals with inducible VTs, 22 viable myocardial channels were detected (an average of 1.5 per animal). Among the 22 viable myocardial channels, 16 myocardial channels were detected as heterogeneous myocardial channels (average of 1.1 per animal). In 5 animals with non-inducible VTs, 3 viable myocardial channels were detected (average 0.6 per animal), which resulted in only 1 HT channel (average 0.2 per animal). HT channels were characterized by heterogeneous myocardium features mingled with scar, typically located along the anterior side of the interventricular groove, at the subepicardium and/or mid-myocardium underneath epicardial fat surrounding the LAD.

**Figure 6:**
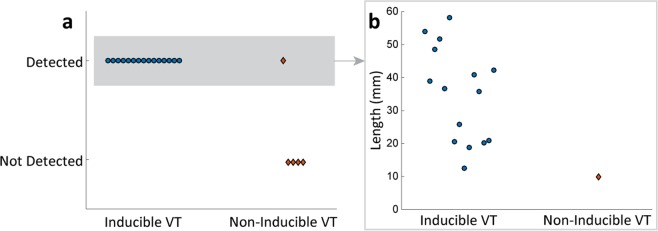
Results of heterogeneous tissue (HT) channel detection. (**a**) HT channel was detected in all animals with inducible ventricular tachycardias (VT), whereas it was only detected in one animal with a non-inducible VT. Animals with inducible VTs were more likely to have HT channels detected than animals with non-inducible VTs (P < 0.01, Fisher’s exact test). (**b**) Of all animals with detected HT channels, only one non-inducible VT animal had a shorter channel length (inducible-VT animals: 35 ± 14 mm vs. non-inducible VT animal: 9.94 mm).

Animals with inducible VTs were more likely to have HT channels than animals with non-inducible VTs (P < 0.01, Fisher’s exact test; Fig. 6a). The HT channel in one non-inducible VT animal was shorter in length compared to those in animals with inducible VTs (inducible-VT animals: 35 ± 14 mm vs. non-inducible VT animal: 9.94 mm; Fig. 6b).

### Site of origin of VT

Among 20 animals with prior myocardial infarction, VTs were induced in 15 animals (75%) by a programmed stimulation or spontaneous premature ventricular contractions. One animal was excluded from the analysis due to the lack of ECG data. A total of 31 monomorphic VTs were induced in 14 swine and the average number of induced VTs was 2.2 per animal. Since previous studies showed that left bundle branch block (LBBB) pattern VT arose from the RV or the interventricular septum (IVS) and right bundle branch block (RBBB) pattern VT arose from the LV^24^, we further analyzed whether the site of VT origin could be related to the location of detected HT channels. The mean cycle length of VTs was 244 ± 38 ms and the duration of QRS was 126 ± 23 ms. 90% (28/31 VTs) had LBBB pattern, while only 10% (3/31 VTs) had RBBB pattern. All animals with LBBB VTs had structural breakthrough sites at the RV or IVS, at the RV apex and the anteroseptum of the mid-LV, corresponding to the distal end and the proximal end of the HT channels, respectively.

**Figure 7:**
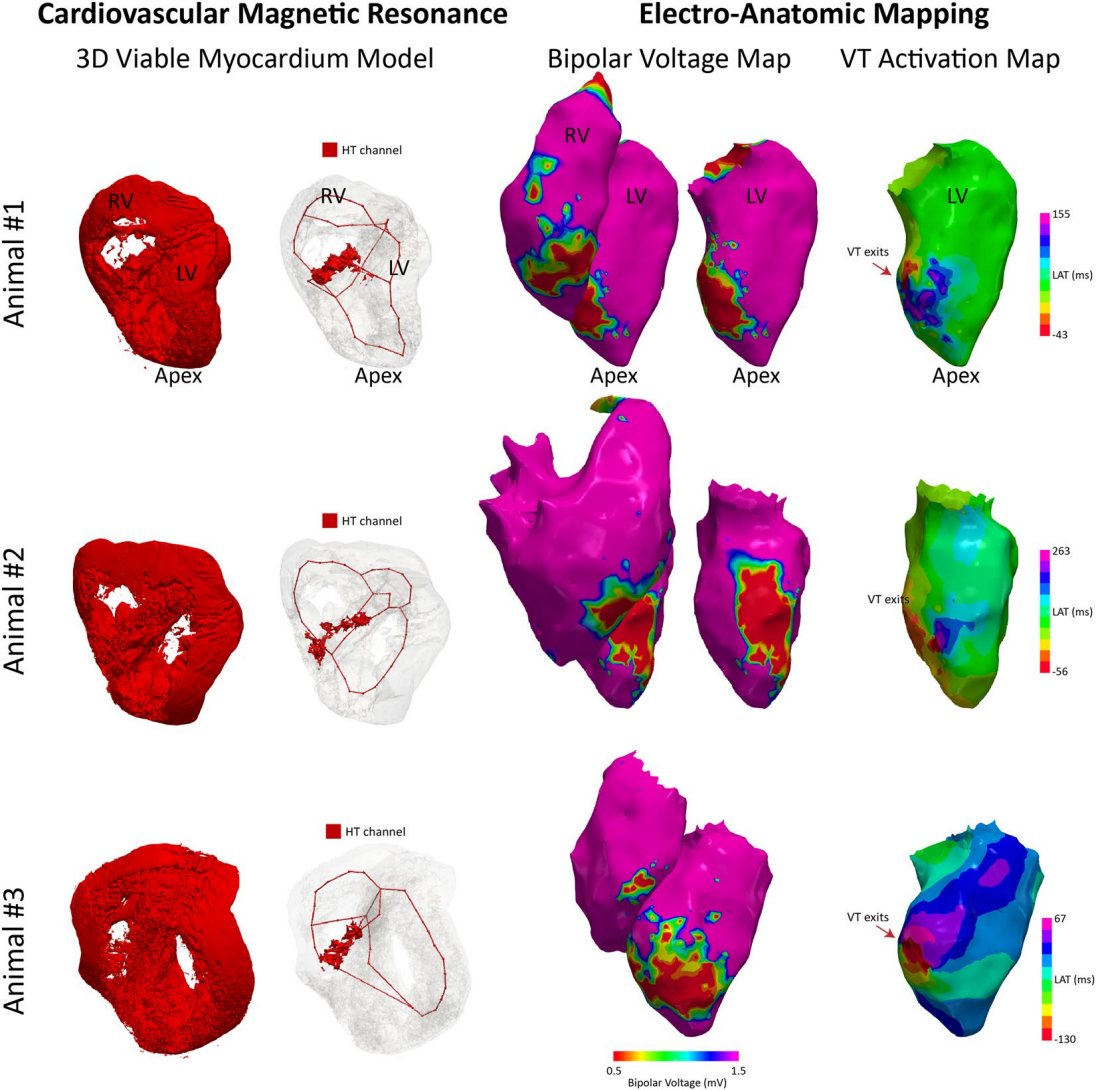
3D viable tissue model and detected HT channels compared to the electroanatomic mapping data in 3 animals with inducible VT. Bipolar voltage maps during sinus rhythm in both right and left ventricles show similar scar distribution compared to the CMR 3D model. VT exits identified in the activation maps during VT are located near the anterior side of interventricular groove, consistent with where HT channels were detected.

More detailed localization of the VT origin was identified in electro-anatomic mapping (EAM) by activation mapping during the VT in 3 animals, which was compared to the location of the detected HT channels. VT exit sites were identified as the earliest activation points. Bipolar voltage maps of both left and right ventricles during the sinus rhythm were compared to localize exit sites on the VT activation maps. For all animals, VT exits were located near the anterior side of the interventricular groove, where HT channels were predominantly detected (Fig. 7), suggesting potential involvement of detected HT channels in the VT.

### Comparison to histology

**Figure 8:**
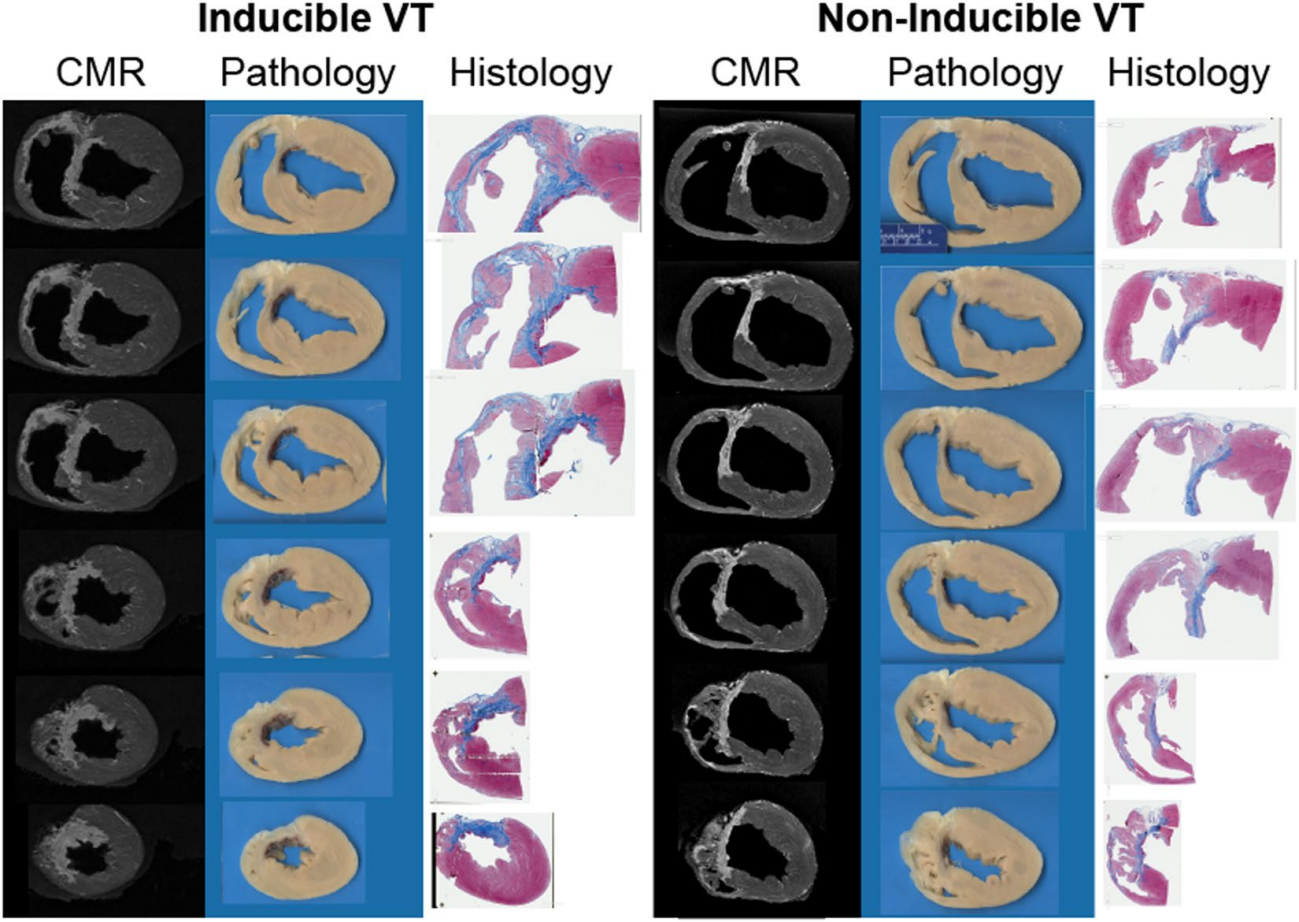
Comparison between CMR and histopathological analyses. A 5-*µ*m histological slide with Masson’s trichrome staining are shown in an anterior-posterior projection. The histological specimen shows complex scar architecture with areas of transmural scar and subendocardial preservation, along with extensive right ventricle and interventricular septum involvement. The overall architecture and distribution of scar were consistent with the LGE data.

Histopathological analysis revealed a complex viable myocardium structure in the anterior wall post-infarction due to the involvement of the RV and IVS. The overall architecture and distribution of scar were consistent with the CMR data (Fig. 8). Analysis of the scar distribution showed heterogeneous distribution of collagen within the infarct and particularly along the anterior side of the interventricular groove, consistent with the typical location of detected HT channels (Fig. 8).

## Discussion

CMR shows promise to characterize the arrhythmogenic substrate. Our study proposes a streamlined workflow to identify HT channels located within areas of dense scarring from high-resolution *ex-vivo* T_1_-weighted CMR images. 3D structural modeling was performed to extract viable myocardium in and around the scar. The 3D skeleton was created to automate characterization of structural properties of the viable myocardium model. Based on the skeleton, the cross-sectional area and tissue heterogeneity were characterized to detect HT channels, which are of interest for causing slow conduction. The proposed technique was successfully tested in 20 animals with prior myocardial infarction, 15 with scar-related VT and 5 without VT. Our results suggest that animals with inducible VT are more likely to have HT channels than animals with non-inducible VTs. We used 12-lead ECG and EAM to identify the site of origin during VT, which showed preference to the LBBB pattern, consistent with the structural breakthrough site observed at the hinge point of HT channels located at the interventricular groove anchored to two different regions of intact healthy myocardium. Histopathological analyses confirmed heterogeneous distribution of the viable tissue along the anterior side of the interventricular groove where HT channels were predominantly detected.

We focused on the modeling of the viable tissue, where electrical signals travel, rather than the scar. 3D modeling of the viable myocardium enables us to understand cardiac impulse propagation in a true 3D manner. 3D skeletonization enables visualization of potential reentrant circuits based on the anatomical structure, reducing physician burden to imagine potential anatomical electrical pathways. Visualizing anatomical circuits may further improve our understanding of structural and anatomical substrates for arrhythmias.

The proposed technique allows automated quantification of 3D structural properties based on segmented skeletal analysis. The 3D skeletal graph consists of nodes and edges, which abstract a shape of the structural model. 3D graphical representation of the structure allows automated quantification of the structural properties of each segment and their connectivity. Therefore the structural characteristics of the model, such as length, area, and thickness can be automatically quantified based on the simplified graph. It may be useful to quantify the length of the anatomical pathway, as reentry is only initiated when the length of the anatomical circuit is greater than the wavelength^26^. Automated quantification of the anatomical isthmus may offer an automated diagnosis of VT vulnerability.

The proposed technique allows automated pinpointing of HT channels and can be applied to any region of the heart to localize any heterogeneous structure or anatomical isthmus that may be susceptible to slow conduction. This technique can be further extended to detect any desired structure with certain structural properties. If merged with electroanatomic data or computer simulation, our understanding of the VT mechanism would be improved and could support personalized planning for VT ablation. Outcome of effective ablation of such structures identified on the proposed technique should be studied further.

Our study has several limitations. The proposed technique in this study is purely an anatomical approach and not applicable to functional mechanisms of arrhythmias. We did not study whether ablation of anatomic regions identified with the proposed technique would improve VT ablation outcome. The correlation between HT channels, the site of VT origin, and histology was made as carefully as possible; however, it is based on gross estimation. The technique was not tested *in-vivo*, although it is expected to be applicable with the advancement of high-resolution *in-vivo* CMR imaging technique. This study focused on macro tissue structures. Future studies will have to account for micro tissue structures by generating the 3D model in higher resolution and performing image segmentation and structural modeling in finer detail.

## Supplementary information

Supplementary Video 1

Supplementary Video 2

Supplementary Information

## Data Availability

The datasets generated and/or analyzed during the current study are available from the corresponding author on reasonable request.

## References

[1] Shah DC (1997). Three-dimensional mapping of the common atrial flutter circuit in the right atrium. Circ..

[2] Ja¨ıs P (2004). Technique and results of linear ablation at the mitral isthmus. Circ..

[3] De Bakker J (1993). Slow conduction in the infarcted human heart.’zigzag’ course of activation. Circ..

[4] Kim RJ (1996). Myocardial gd-dtpa kinetics determine mri contrast enhancement and reflect the extent and severity of myocardial injury after acute reperfused infarction. Circ..

[5] Kim RJ (1999). Relationship of mri delayed contrast enhancement to irreversible injury, infarct age, and contractile function. Circ..

[6] Schmidt A (2007). Infarct tissue heterogeneity by magnetic resonance imaging identifies enhanced cardiac arrhythmia susceptibility in patients with left ventricular dysfunction. Circ..

[7] Ferna´ndez-Armenta J (2012). Use of myocardial scar characterization to predict ventricular arrhythmia in cardiac resynchronization therapy. Eur..

[8] Scott Paul A., Morgan John M., Carroll Nicola, Murday David C., Roberts Paul R., Peebles Charles R., Harden Stephen P., Curzen Nick P. (2011). The Extent of Left Ventricular Scar Quantified by Late Gadolinium Enhancement MRI Is Associated With Spontaneous Ventricular Arrhythmias in Patients With Coronary Artery Disease and Implantable Cardioverter-Defibrillators. Circulation: Arrhythmia and Electrophysiology.

[9] Roes SD (2009). Infarct tissue heterogeneity assessed with contrast-enhanced mri predicts spontaneous ventricular arrhythmia in patients with ischemic cardiomyopathy and implantable cardioverter-defibrillatorclinical perspective. Circ. Cardiovasc. Imaging.

[10] Ashikaga H (2007). Magnetic resonance–based anatomical analysis of scar-related ventricular tachycardia. Circ. research.

[11] Estner HL (2011). The critical isthmus sites of ischemic ventricular tachycardia are in zones of tissue heterogeneity, visualized by magnetic resonance imaging. Hear. Rhythm..

[12] Dickfeld T (2011). Mri-guided ventricular tachycardia ablation. Circ. Arrhythmia Electrophysiol..

[13] Perez-David E (2011). Noninvasive identification of ventricular tachycardia-related conducting channels using contrast- enhanced magnetic resonance imaging in patients with chronic myocardial infarction: comparison of signal intensity scar mapping and endocardial voltage mapping. J. Am. Coll. Cardiol..

[14] Andreu David, Berruezo Antonio, Ortiz-Pérez José T., Silva Etelvino, Mont Lluis, Borràs Roger, de Caralt Teresa María, Perea Rosario Jesús, Fernández-Armenta Juan, Zeljko Hrvojka, Brugada Josep (2011). Integration of 3D Electroanatomic Maps and Magnetic Resonance Scar Characterization Into the Navigation System to Guide Ventricular Tachycardia Ablation. Circulation: Arrhythmia and Electrophysiology.

[15] Fernández-Armenta Juan, Berruezo Antonio, Andreu David, Camara Oscar, Silva Etelvino, Serra Luis, Barbarito Valeria, Carotenutto Luigi, Evertz Reinder, Ortiz-Pérez José T., De Caralt Teresa María, Perea Rosario Jesús, Sitges Marta, Mont Lluis, Frangi Alejandro, Brugada Josep (2013). Three-Dimensional Architecture of Scar and Conducting Channels Based on High Resolution ce-CMR. Circulation: Arrhythmia and Electrophysiology.

[16] Andreu D (2017). Cardiac magnetic resonance–aided scar dechanneling: Influence on acute and long-term outcomes. Hear. Rhythm..

[17] Fedorov A (2012). 3d slicer as an image computing platform for the quantitative imaging network. Magn. resonance imaging.

[18] Cao, J., Tagliasacchi, A., Olson, M., Zhang, H. & Su, Z. Point cloud skeletons via laplacian based contraction. In *Shape Modeling International Conference (SMI)*, *2010*, 187–197 (IEEE, 2010).

[19] Au OK-C, Tai C-L, Chu H-K, Cohen-Or D, Lee T-Y (2008). Skeleton extraction by mesh contraction. ACM Transactions on Graph. (TOG).

[20] Valsangiacomo Buechel ER, Mertens LL (2012). Imaging the right heart: the use of integrated multimodality imaging. Eur. heart journal.

[21] Galea N (2013). Right ventricular cardiovascular magnetic resonance imaging: normal anatomy and spectrum of pathological findings. Insights into imaging.

[22] Tschabrunn CM (2016). A swine model of infarct-related reentrant ventricular tachycardia: electroanatomic, magnetic resonance, and histopathological characterization. Hear. Rhythm..

[23] Whitaker, J. *et al*. Cardiac mr characterization of left ventricular remodeling in a swine model of infarct followed by reperfusion. *J*. *Magn*. *Reson*. *Imaging* (2018). 10.1002/jmri.26005 29522262

[24] Josephson ME (1981). Sustained ventricular tachycardia: role of the 12-lead electrocardiogram in localizing site of origin. Circ..

[25] Josephson ME, Callans DJ (2005). Using the twelve-lead electrocardiogram to localize the site of origin of ventricular tachycardia. Hear. Rhythm..

[26] Zimetbaum, P. J. & Josephson, M. E. *Practical clinical electrophysiology* (Lippincott Williams & Wilkins, 2017).

