# Supplementary material for: Cardiovascular Magnetic Resonance-Based Three-Dimensional Structural Modeling and Heterogeneous Tissue Channel Detection in Ventricular Arrhythmia
Source: Sci Rep. 2019 Jun 27;9:9317. doi: 10.1038/s41598-019-45586-1 (PMC6597699; doi:10.1038/s41598-019-45586-1)
Supplement: Supplementary file 3 — Supplementary Information [file 41598_2019_45586_MOESM3_ESM.docx]

**Supplementary Information**

**Cardiovascular Magnetic Resonance-Based Three-Dimensional Structural Modeling and Heterogeneous Tissue Channel Detection in Ventricular Arrhythmia**

Jihye Jang^1,2^, Hye-Jin Hwang^1^, Cory M. Tschabrunn^1,3^, John Whitaker^1,4^, Bjoern Menze^2^,

Elad Anter^1^, Reza Nezafat^1^

**Affiliations:**

^1^Department of Medicine, Beth Israel Deaconess Medical Center and Harvard Medical School, Boston, MA, USA.

^2^Department of Computer Science, Technical University of Munich, Munich, Germany.

^3^Division of Cardiovascular Medicine, University of Pennsylvania, Philadelphia, PA, USA.

^4^Division of Imaging Sciences and Biomedical Engineering, King’s College London, London, United Kingdom.

*Corresponding author: Reza Nezafat, [rnezafat@bidmc.harvard.edu](mailto:rnezafat@bidmc.harvard.edu).


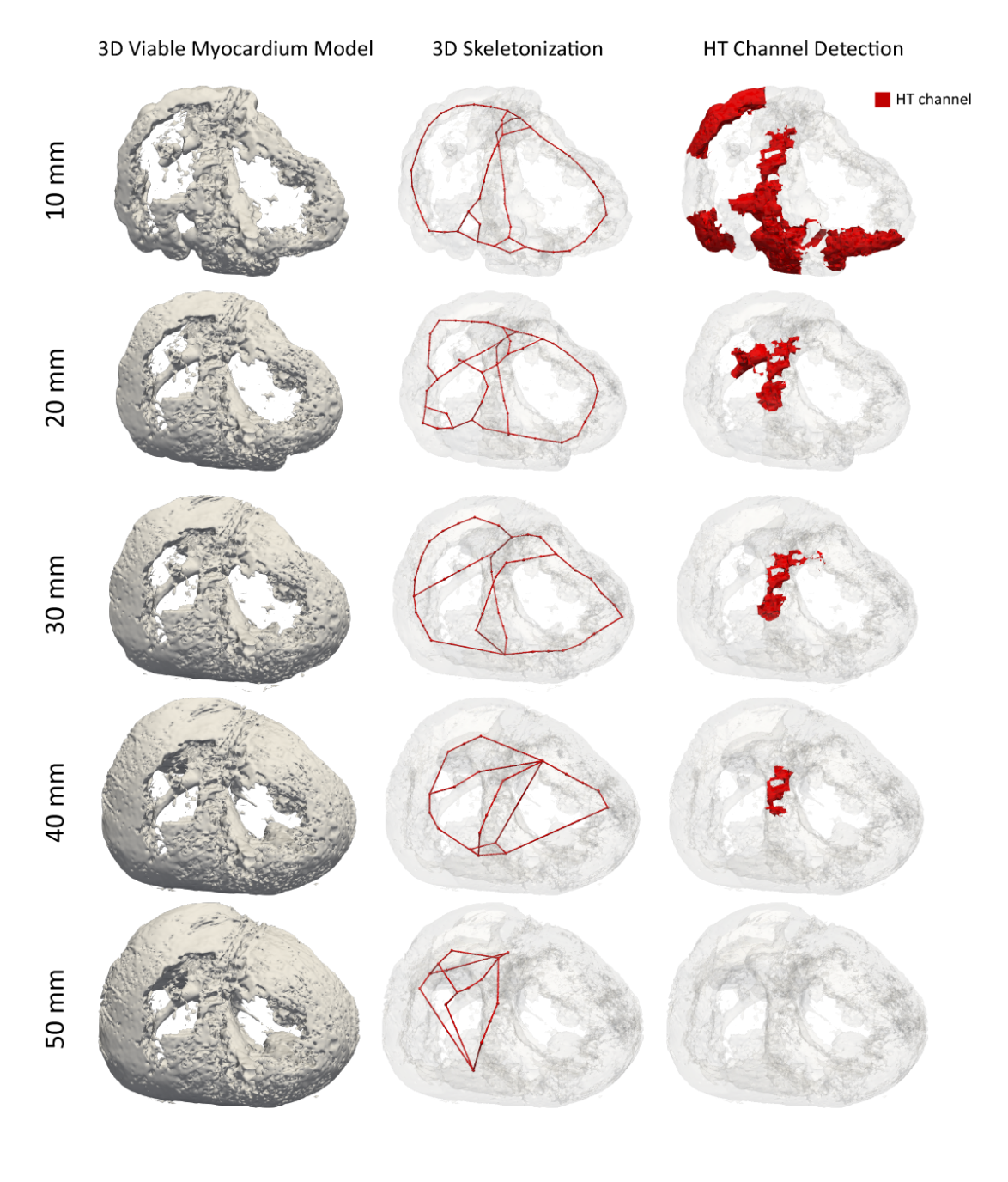


**Supplementary Figure 1.** Diameters of the myocardial volume surrounding the scar alongside their corresponding 3D skeletonization and heterogeneous tissue (HT) channel detection results. The shorter the diameter, the easier it was to perform 3D skeletonization. The longer the diameter, the easier it was to discriminate thin tissue channels from normal left/ right ventricle, and septal wall. We selected 30 mm satisfying compromise between skeletonization and channel discrimination, and used it throughout the study.

*
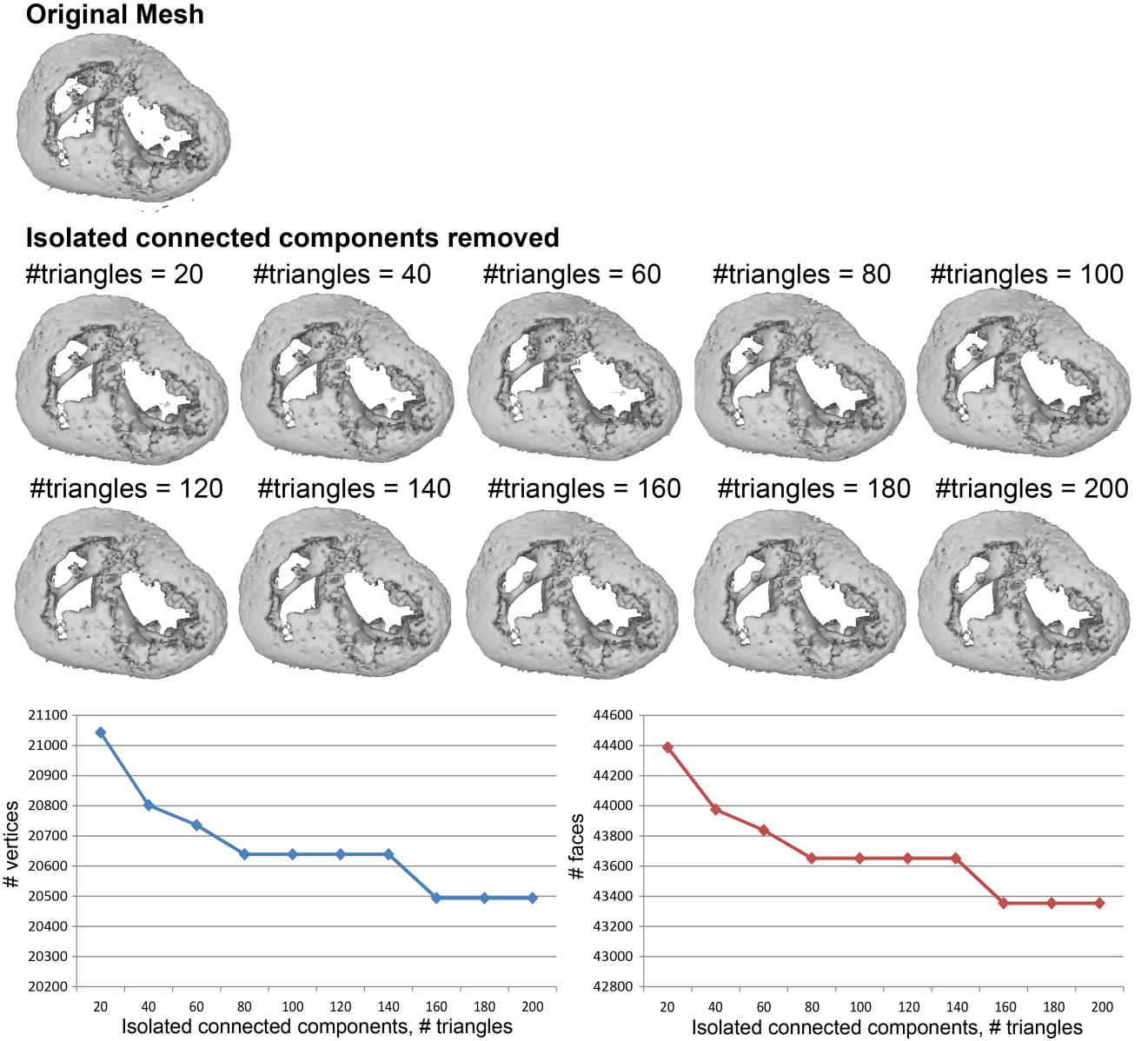
*

**Supplementary Figure 2**. Isolated connected components composed by a limited number of triangles were representative of noise. We show 3D viable tissue models upon removal of isolated connected components with varying number of triangles. Both the number of vertices and the number of faces of the model initially flattened at 80 triangles, then at 160 triangles. The minimum number of triangles necessary to denoise the model was set to 100.


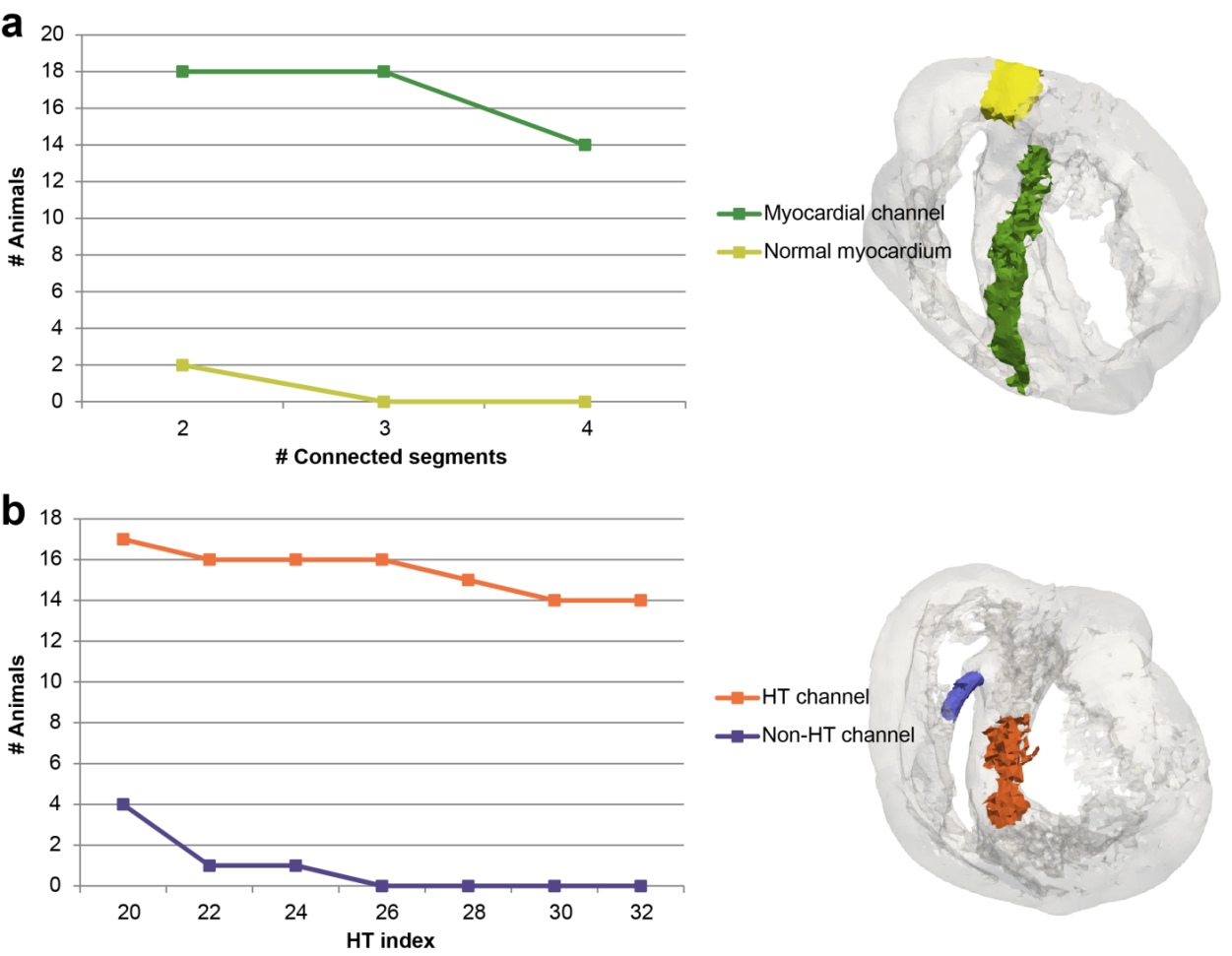


**Supplementary Figure 3**. a) The number of animals with accurately detected myocardial channels vs. falsely detected normal myocardium segments (left/ right ventricle, and septum) depending on the minimum number of connected segments. b) The number of animals with accurately detected heterogeneous tissue (HT) channels vs. smooth tissue channels (Non-HT channel; i.e. moderator band) depending on the HT index.


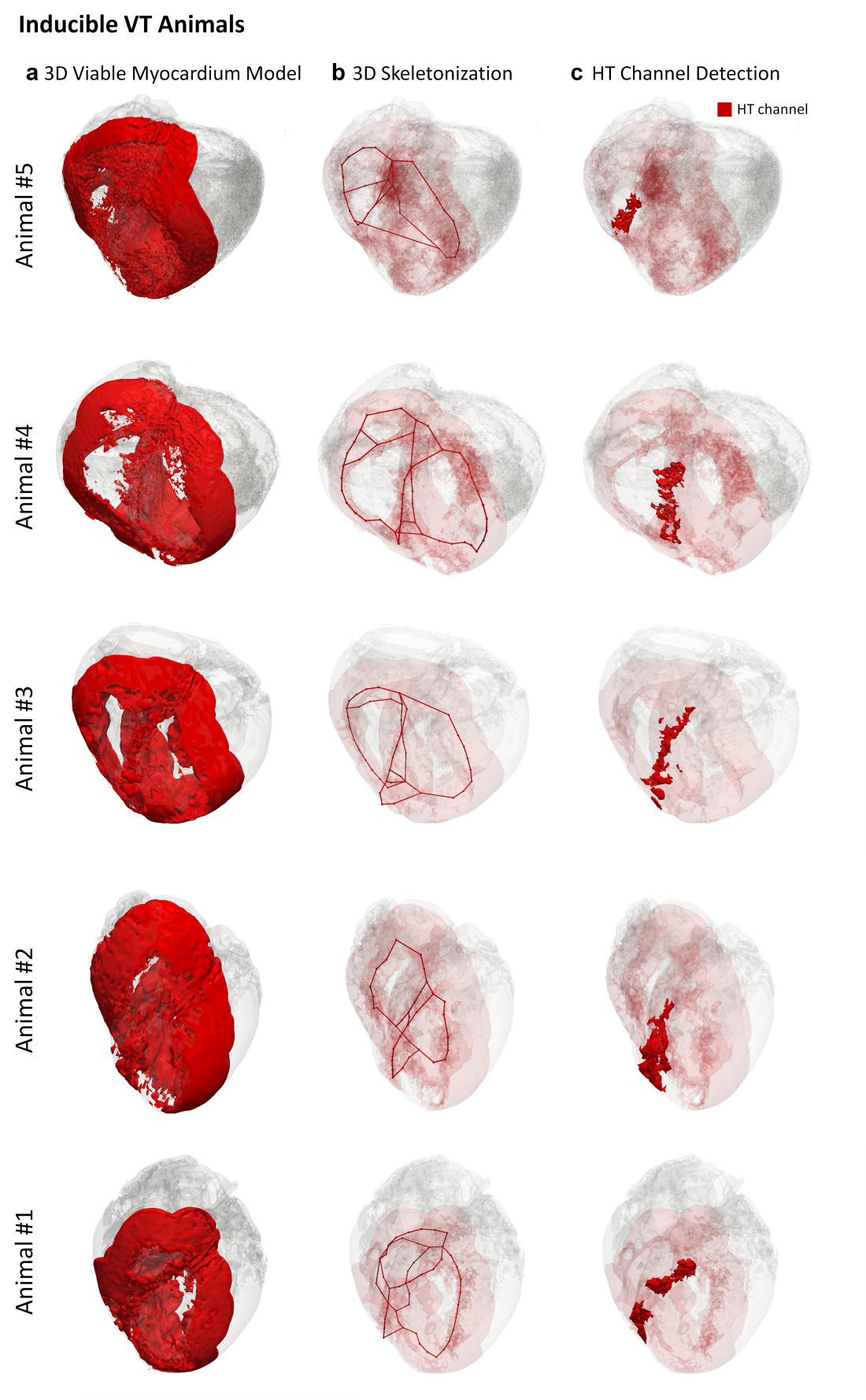


**Supplementary Figure 4.** Results of all 15 inducible VT animals (Animal#1 – Animal#5).


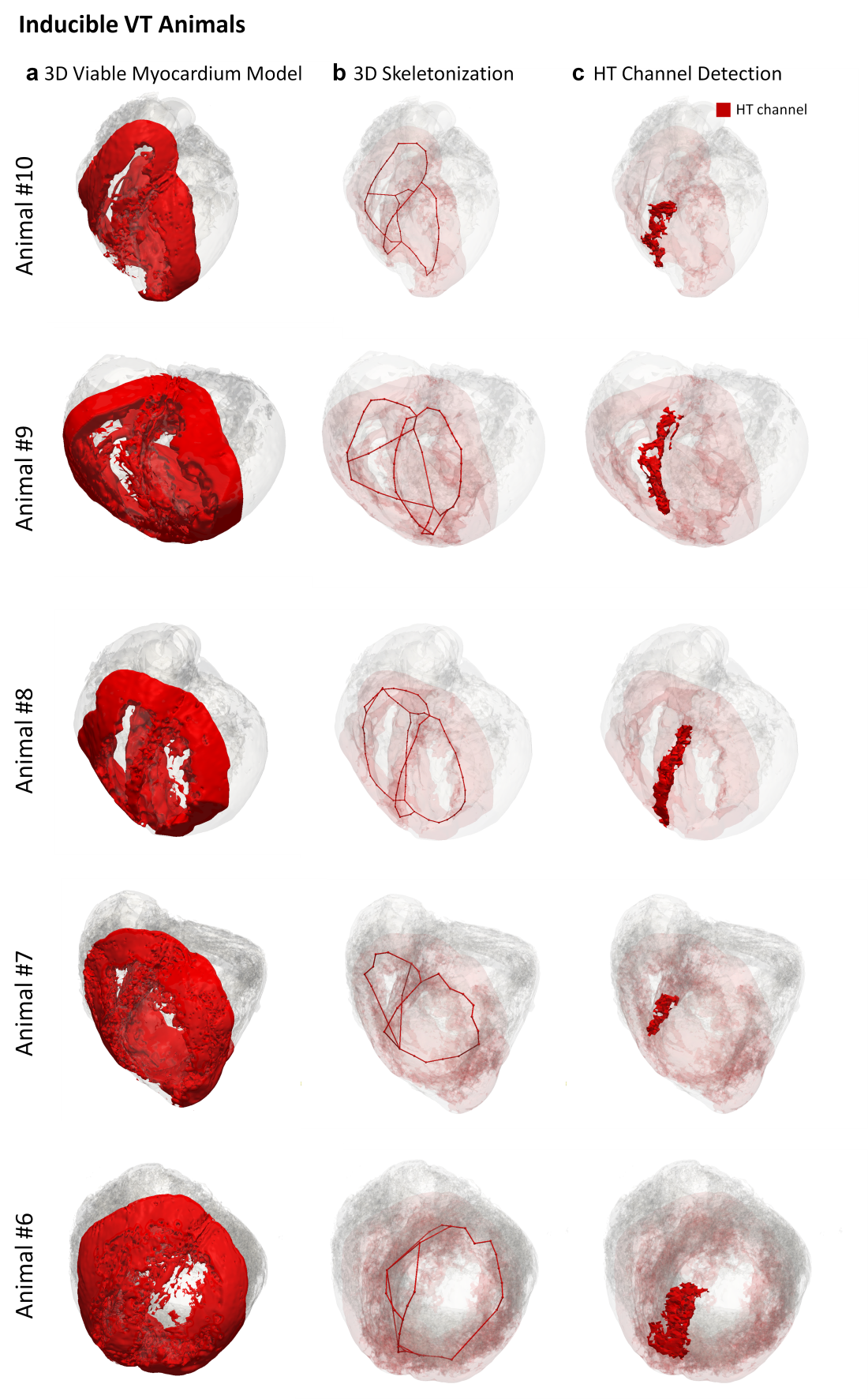


**Supplementary Figure 5.** Results of all 15 inducible VT animals (Animal#6 – Animal#10).


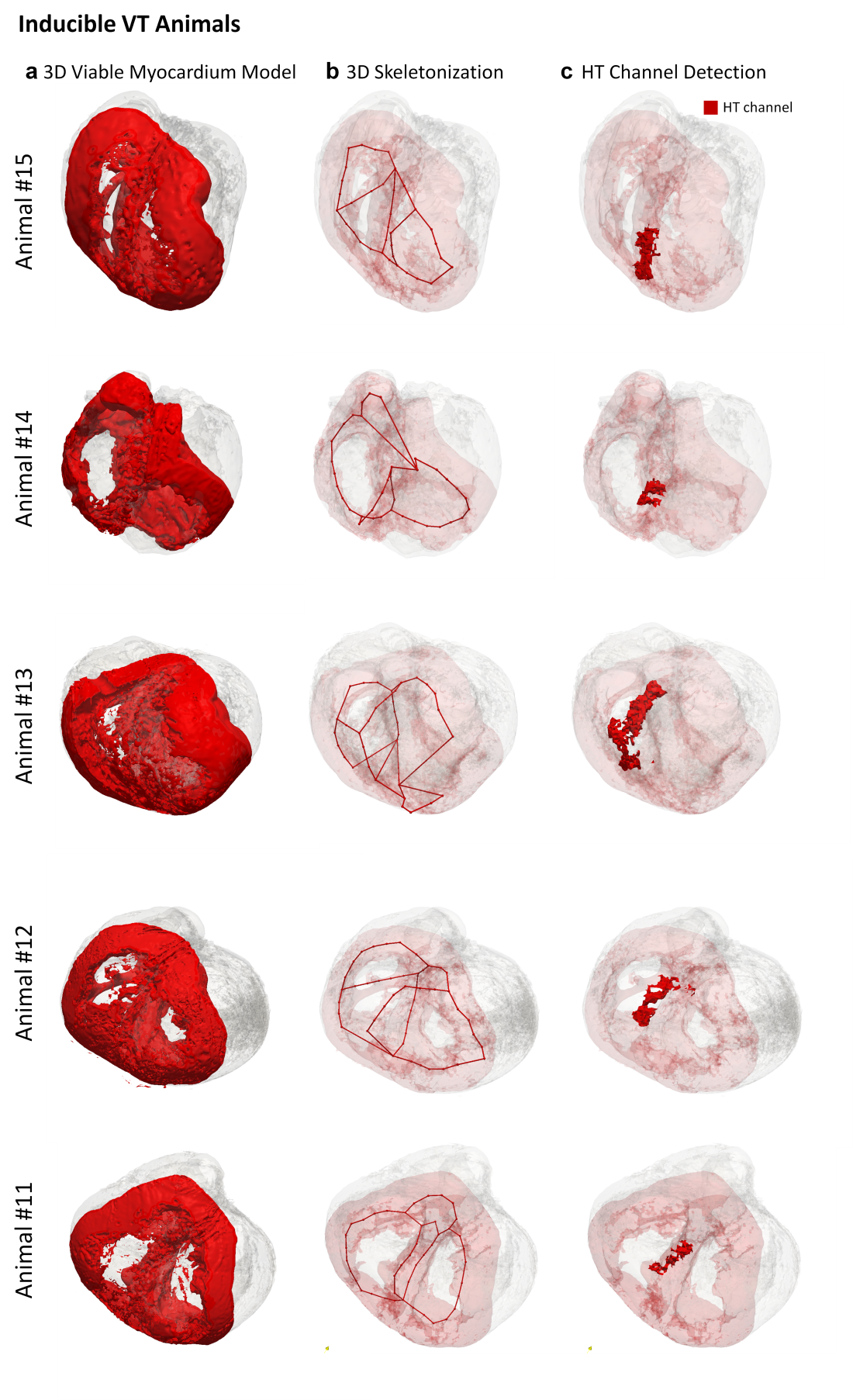


**Supplementary Figure 6.** Results of all 15 inducible VT animals (Animal#11 – Animal#15).


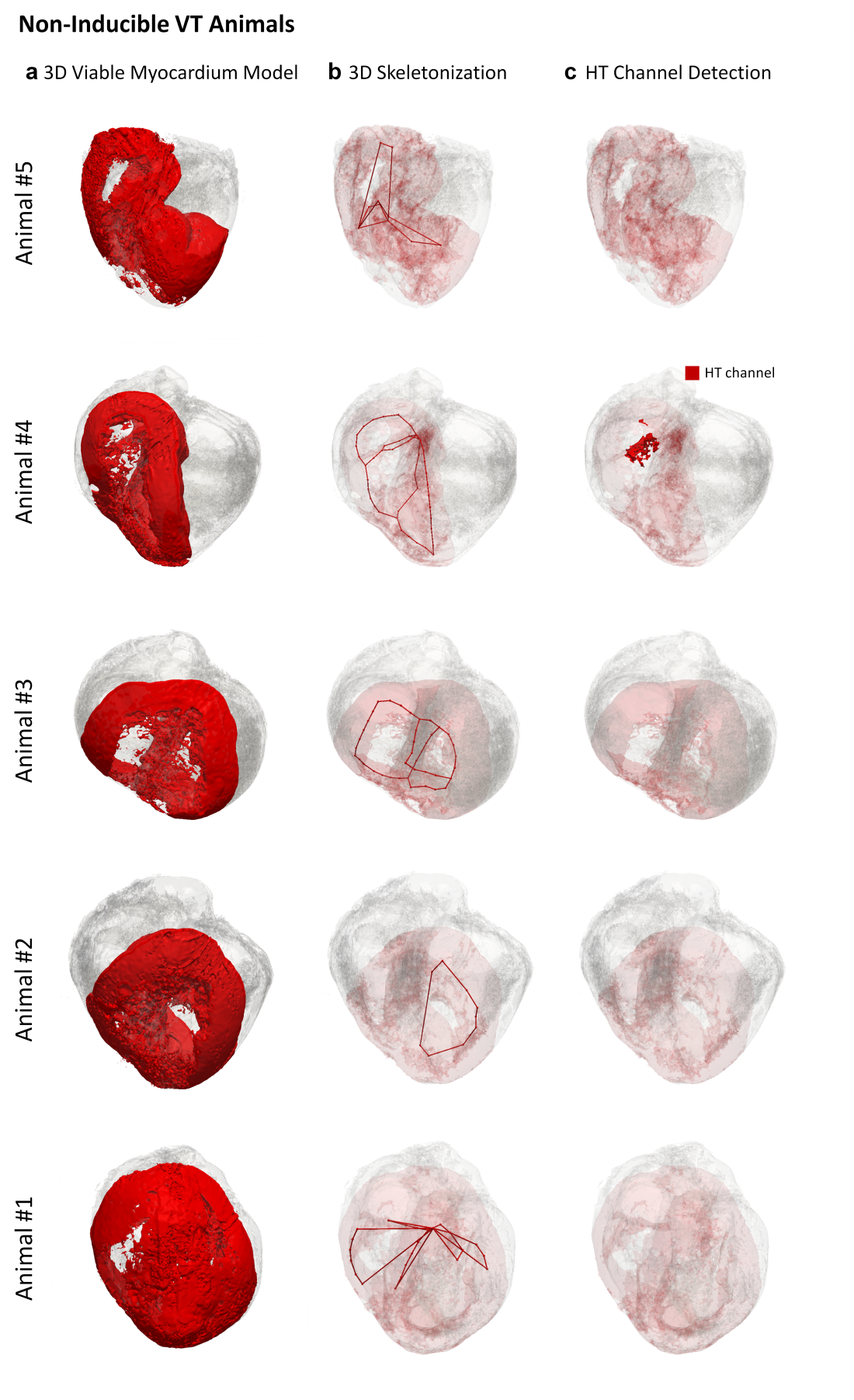


**Supplementary Figure 7.** Results of all 5 non-inducible VT animals (Animal#1 – Animal#5).


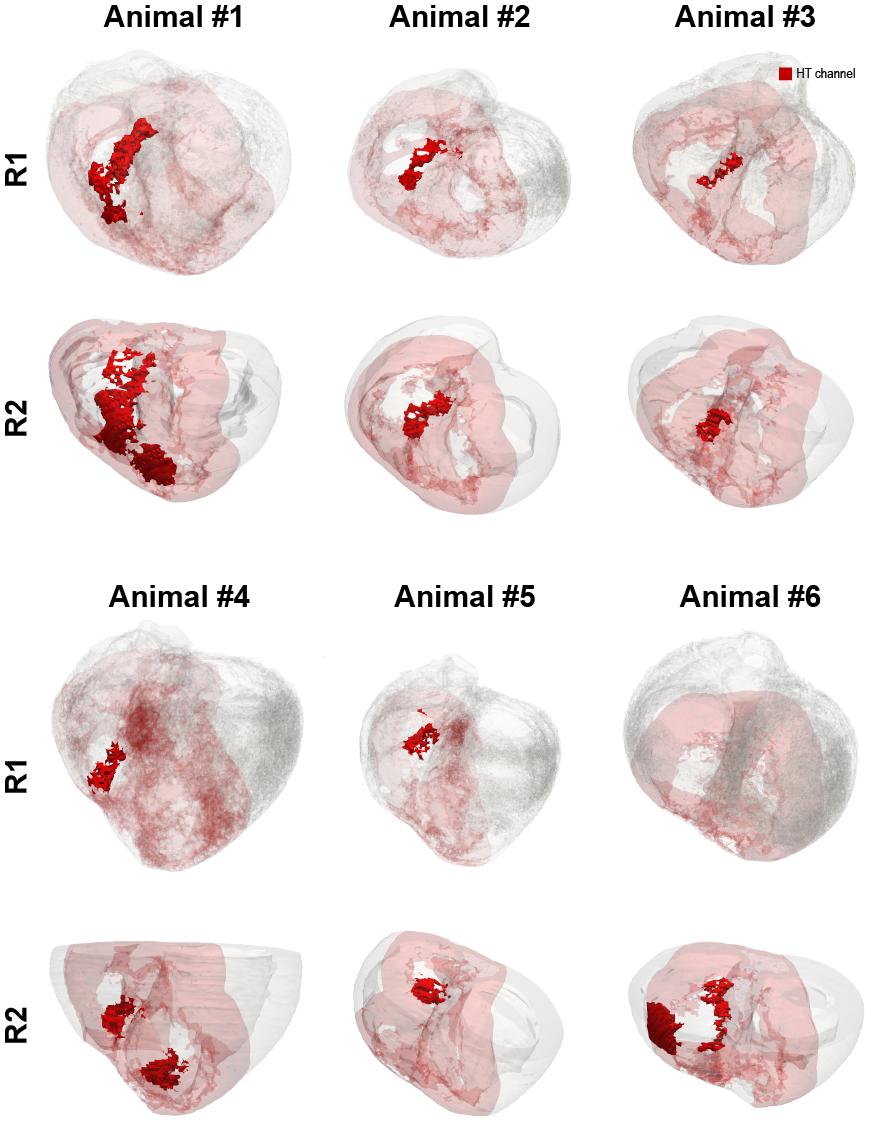


**Supplementary Figure 8.** Inter-observer analysis between two independent readers (R1 vs. R2). Two readers independently performed scar/myocardium segmentation, and the proposed technique was applied on these segmentations. Inter-observer agreement between two independent readers was strong (Dice index for scar segmentation of 0.79 ± 0.07). Similar heterogeneous tissue (HT) channel detection results were shown in 5 out of 6 animals.
